# Influencing of serum inflammatory factors on IVF/ICSI outcomes among PCOS patients with different BMI

**DOI:** 10.3389/fendo.2023.1204623

**Published:** 2023-08-24

**Authors:** Yilei He, Rong Li, Jingwen Yin, Zi Yang, Yuanyuan Wang, Lixue Chen, Shuo Yang, Jie Qiao

**Affiliations:** ^1^ Center for Reproductive Medicine, Department of Obstetrics and Gynecology, Peking University Third Hospital, Beijing, China; ^2^ National Clinical Research Center for Obstetrics and Gynecology, Peking University Third Hospital, Beijing, China; ^3^ Key Laboratory of Assisted Reproduction, Peking University, Ministry of Education, Beijing, China; ^4^ Beijing Key Laboratory of Reproductive Endocrinology and Assisted Reproductive Technology, Beijing, China

**Keywords:** body mass index, inflammatory factor, polycystic ovary syndrome, *in vitro* fertilization/intracytoplasmic sperm injection, pregnancy outcomes

## Abstract

**Introduction:**

Overweight and obese are important factors leading to the occurrence of long-term complications in women with polycystic ovary syndrome (PCOS). There has been controversy over whether dissatisfaction with pregnancy outcomes in PCOS patients is influenced by chronic inflammatory status or obesity. This retrospective study analyzed the levels of inflammatory factors in PCOS patients with different body mass index (BMI) groups and effective predictors of *in vitro* fertilization/intracytoplasmic sperm injection (IVF/ICSI) pregnancy outcomes.

**Methods:**

There were 273 women with PCOS diagnosed who completed serum inflammatory factors test between January 2017 and June 2022 were selected. The data of 7,649 infertility PCOS patients who received their first IVF/ICSI treatment in the Reproductive Center of Peking University Third Hospital during the period of the study were collected. Finally, 92 PCOS patients were included in the high BMI group, while 97 patients were included in the normal BMI group. Baseline characteristics were collected and the pregnancy outcomes were compared among the two groups. Then, serum inflammatory factors’ effect on IVF/ICSI pregnancy outcomes were analyzed with age, anti-Mullerian Hormone (AMH) and BMI adjusted.

**Results:**

PCOS patients in the high BMI group significantly had a lower number of oocytes retrieved and good quality embryos. The high BMI group PCOS patients had higher levels of IL-6 and lower cumulative clinical pregnancy and live birth rates. The level of GM-CSF was higher in the first cycle transfer and cumulative miscarriage group. High TNF-α was negatively correlated with the first transfer cycle and cumulative clinical pregnancy rates after age, AMH and high BMI adjusted. In addition, the cumulative live birth rate was negatively correlated with high IL-6, but the first cycle transfer and cumulative live birth rates were positively correlated with high IL-1β.

**Discussion:**

For PCOS patients, in addition to BMI, attention should also be paid to inflammatory indicators. High levels of TNF-α and IL-6 were negatively correlated with pregnancy outcomes, but high IL-1β was positively correlated with live birth rates among PCOS patients. The level of GM-CSF was higher in miscarriage PCOS patients.

## Introduction

Polycystic ovary syndrome (PCOS) is a common infertility-related disease caused by endocrine and metabolic abnormalities in women of childbearing age. The main manifestations are oligomenorrhea/amenorrhea, polycystic ovarian changes, and hyperandrogenism. During the last decade, the public health problems like overweight and high blood pressure were more obvious. Life stress and working pressure increased, with an elevation in the incidence of oligo/amenorrhea and hyperandrogenism among childbearing-age women. On the other hand, in recent years, the further deepening of Chinese people’s awareness of PCOS and the expansion of the detection population were also one of the reasons for the increase in the incidence of PCOS, which has reached 10.01% in China at present (1). PCOS often manifests with chronic inflammation status and obesity. High body mass index (BMI) can affect oocyte development, fertilization rate, and embryo quality, leading to pregnancy failure (2). A multicenter, prospective, observational study of Chinese PCOS patients, illustrated the association between elevated BMI and reduced clinical pregnancy rate, but similar ongoing pregnancy rates were observed among patients with different BMI groups (3).

Chronic low-grade inflammation is a well-described feature of both obesity and the risk of adverse outcomes in obesity-associated diseases. Compared with age and BMI-matched control groups, the C-reaction protein, interleukin (IL)-1β, IL-18, white blood cell, monocyte chemoattractant protein-1, and macrophage inflammatory protein-1α levels were elevated in women with PCOS (4). Along with having higher levels of oxidative stress, women with PCOS have lower levels of antioxidant proteins as well as a minor total antioxidant status (5). Inflammatory factors play a role in a range of female reproductive processes, including ovulation, follicular increase, fertilization, and successful pregnancy as Brouillet S, et al. mentioned in their systematic review (6). Some studies showed that lymphocytes and macrophages in ovarian tissue of patients with PCOS were higher than those of normal people, and a large amount of tumor necrosis factor-alpha (TNF-α), IL-6, et al. were secreted, which activated NF-κB, leading to premature apoptosis of granulosa cells and inhibiting the formation of dominant follicles (7), affecting ovulation and pregnancy. However, there has been controversy over whether dissatisfaction with pregnancy outcomes in PCOS patients is influenced by chronic inflammatory status or obesity.

Given that chronic nonspecific inflammatory factors play a role in both obesity and the onset of PCOS, we present a retrospective cohort study that analyzed the levels of inflammatory factors in PCOS patients with different BMI groups and effective predictors of *in vitro* fertilization (IVF)/intracytoplasmic sperm injection (ICSI) outcomes.

## Methods

### Subjects

We collected clinical data from patients with PCOS who received their first IVF/ICSI with standard ovarian stimulation treatment and all the embryos transferred within two years in the Reproductive Center of Peking University Third Hospital between January 2017 and June 2022.

Inclusion criteria: 1. Age between 19 to 44 years old; 2. Diagnosed PCOS according to the “Rotterdam Criteria” (8); 3. Completed serum inflammatory factors test; 4. BMI ≥ 18.5 kg/m^2^.

Exclusion criteria: 1. Patients who were diagnosed with autoimmune diseases, preimplantation genetic test cycle, sperm/oocyte donation cycle, history of recurrent spontaneous abortion; 2. Patients with uncontrolled hyperprolactinemia and thyroid dysfunction; 3. Patients with uterine malformation, submucosal myoma, intrauterine adhesions, and untreated hydrosalpinx that affect embryo implantation.

**Figure 1:**
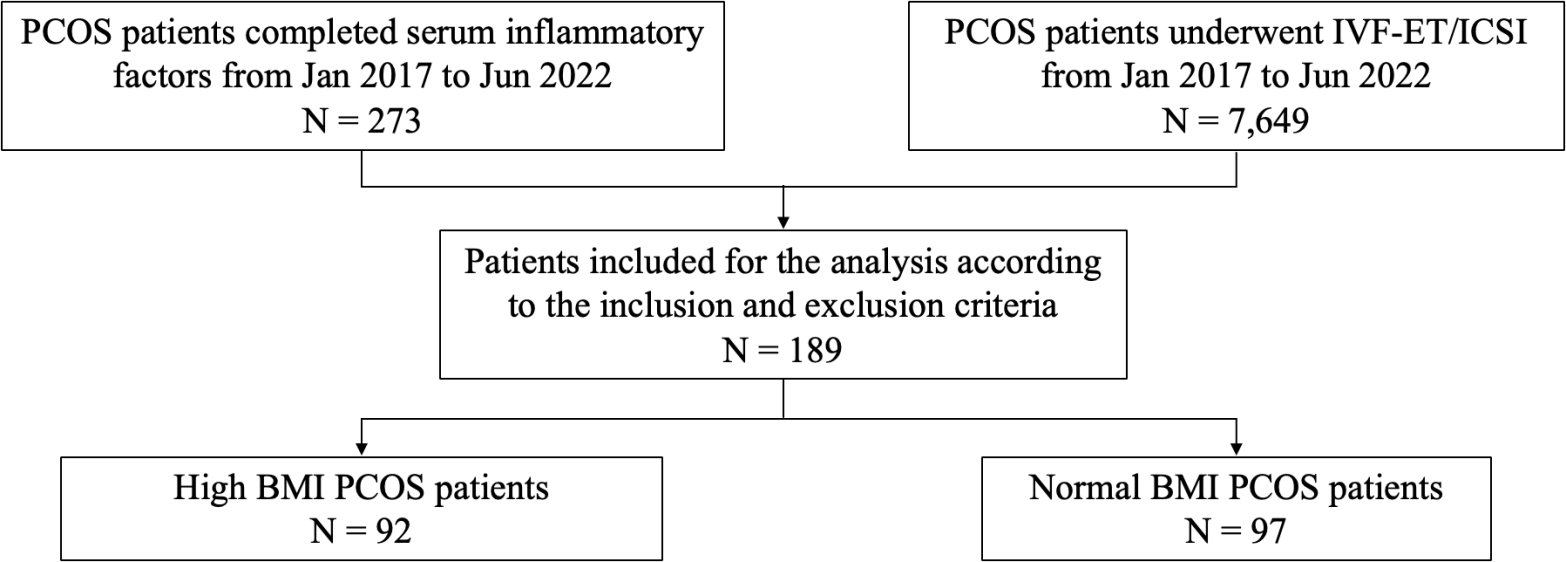
Flow chart of study cohort selection. PCOS, polycystic ovary syndrome; IVF/ICSI, *in vitro* fertilization/intracytoplasmic sperm injection; BMI, body mass index.

A total of 273 PCOS patients who completed serum inflammatory factors tests between January 2017 and June 2022 were selected. The data of 7,649 PCOS patients who received IVF/ICSI during the period of the study were collected. Among these PCOS patients, 189 women satisfied the inclusion criteria. Based on BMI (9), PCOS patients were divided into two groups: 1. High BMI group: BMI ≥ 24 kg/m^2^; 2. Normal BMI group: 18.5 kg/m^2^ ≤ BMI < 24 kg/m^2^ (**Figure 1**).

### Definition of groups

After 4 weeks of embryo transfer, vaginal ultrasound examination revealed the presence of a gestational sac, regardless of whether there was fetal heartbeat, indicating a clinical pregnancy. Miscarriage was defined as the loss of clinical pregnancy before 28 weeks of gestation. The delivery of any viable neonate at 28 weeks of pregnancy or above was considered a live birth. The cumulative clinical pregnancy/live birth rate of each group was the number of patients who had at least one gestational sac/live-birth baby after the transfer of all fresh and frozen-thawed/warmed embryos in their first ovarian stimulation cycle divided the number of patients who received ovarian stimulation. The cumulative miscarriage rate referred to the number of patients who had at least one loss of clinical pregnancy before 28 weeks of gestation after transplanting all fresh and frozen-thawed/warmed embryos in the first ovarian stimulation cycle divided the number of clinical pregnancy patients in each group. Severe OHSS was defined according to Navot et al. (10) criteria.

The reproductive center ethics committee of Peking University Third Hospital approved this study, No. 2019SZ-041.

### Sample collection

PCOS patients with regular menstruation did blood analysis on the 2nd day of menstruation, and patients with oligo/amenorrhea did blood analysis at any time. The patient’s the serum levels of Follicle Stimulating Hormone (FSH), Luteinizing Hormone (LH), Estradiol (E_2_), Testosterone (T), Androgen and anti-Mullerian Hormone (AMH) were tested by Immulite 2000 (Los Angeles, California, USA) at the Reproductive Endocrinol Laboratory of Peking University Third Hospital (10).

Cytokines were all tested before ovulation induction at the Hematology Research Laboratory of Peking University Third Hospital. IL-1β, IL-2, IL-4, IL-6, IL-9, IL-10, IL-17, IL-12p70, TNF-α, interferon-alpha (IFN-α), and IFN γ, were measured by the Human Th1/Th2 Cytokine Cytometric Bead Array (CBA) Kit II (Bioscience, Franklin Lakes, NJ, USA, 551809). Serum granulocyte colony‐stimulating growth factor (G‐CSF) and granulocyte monocyte colony‐stimulating growth factor (GM‐CSF) concentrations were measured using another commercially available ELISA kit (PeproTech).

### IVF/ICSI-ET

IVF/ICSI-ET was performed according to the routine procedure of our center (11). Gonadotropins (Gn) including recombinant FSH (Merck Serono, Switzerland), urogenic FSH (Institut biochimique SA, Switzerland) and urogenic human Menopausal Gonadotropin (hMG) (Lizhu Pharmaceutical Co., Zhuhai) were initiated on the second day of menstruation bleeding. After 4-5 days injection of Gn, the serum levels of LH, E_2_, and progesterone were tested, and the growth of follicles were checked by vaginal B-ultrasound. Human chorionic gonadotropin (r-hCG, Adze, 250ug) was applied that night, when three follicles ≥ 18mm (follicles ≥ 17mm for the antagonist regimen) were detected. With the guidance of transvaginal ultrasound, oocytes retrieval was performed 36-38h later. Luteal support and 9% vaginal progesterone gel (Swiss Merck Serono) were given to all patients. For the fresh cycle transfer, one or two embryos would be transferred 3/5 days after the oocyte retrieval if the patient was available, and the remaining embryos were cryopreserved. For the frozen cycle transfer, the endometrial preparation was carried out according to the diagnosis and treatment routine of our center (11). After the endometrial thickness of 8 mm monitored by ultrasound, progesterone 40 mg/day and vaginal progesterone gel 90 mg/day were performed which was set as D0. The embryo was transferred on D3/D5.

### Statistical analysis

Data was processed and analyzed using SPSS 26.0 software. Normally distributed data was expressed as the mean ± standard deviation (SD) and non-normally distributed data was expressed as the median (interquartile range). Categorical data was presented as the percentage (number of cases). The mean between the normal distribution and homogeneity of variance groups was compared using Student’s t-test or one-way ANOVA, otherwise, using the Mann-Whitney U test. Chi-square and Fisher exact tests were used to evaluate the categorical variables between different groups. Linear regression model was conducted to analyze the association between the number of oocytes retrieved and related factors. After adjusting for relevant factors, logistic regression analysis was performed to calculate the odds ratios (ORs) with 95% confidence intervals (CIs). *P* < 0.05 was considered a statistically significant difference and all tests were two-sided.

## Results

As shown in **Table 1**, there were no significant differences between the two groups in age, rate of primary infertility, testosterone, androgen, and ovarian PCO; however, significantly lower LH/FSH and AMH levels were observed in the high BMI group than in the normal BMI group (*P* = 0.004, *P* = 0.002). The hCG day E_2_ level was lower, and Gn days were longer and dose higher in the high BMI group (*P* = 0.009, *P* = 0.004, *P* < 0.001). The normal BMI group had a significantly higher number of oocytes retrieved (17 *vs.* 14, *P* = 0.024), and a higher number of good quality embryos (7 *vs.* 5, *P* = 0.014) than the high BMI group. The incidence of severe OHSS was not significantly different (*P* = 0.194).

**Table 1:** Baseline characteristics and data of controlled ovarian stimulation of PCOS patients.

	High BMI (n=92)	Normal BMI (n=97)	*P*
	mean ± SD/median (IQR)	mean ± SD/median (IQR)	
Age (year)	31.62 ± 3.68	31.57 ± 3.38	0.919
Primary infertility (%)	63.04% (58/92)	61.86% (60/97)	0.866
BMI (kg/m^2^)	27.13 ± 2.53	21.20 ± 1.83	<0.001
LH/FSH	0.76 (0.50-1.32)	1.07 (0.72-1.56)	0.004
T (nmol/L)	0.71 (0.69-1.26)	0.77 (0.69-1.32)	0.392
Androgen (nmol/L)	8.26 (6.41-12.95)	10.08 (7.13-14.35)	0.216
AMH (ng/mL)	5.91 (4.02-8.86)	8.73 (5.83-12.50)	0.002
Ovarian PCO (%)	97.83% (90/92)	94.85% (92/97)	0.278
Protocol of controlled ovarian hyperstimulation (%)			0.148
Long GnRH agonist	7.61% (7/92)	2.06% (2/97)	
Ultralong GnRH agonist	10.87% (10/92)	6.19% (6/97)	
GnRH antagonist	78.26% (72/92)	90.72% (88/97)	
Short GnRH agonist	3.26% (3/92)	1.03% (1/97)	
hCG day E_2_ (pmol/L)	9245.50 (5161.00-15875.00)	12397.00 (8355.00-18017.00)	0.009
hCG day LH (mIU/mL)	1.93 (0.81-3.42)	1.70 (0.91-2.96)	0.630
hCG day P (nmol/L)	1.79 (1.18-3.34)	2.00 (1.49-2.96)	0.382
Gn initial dose (U)	150.00 (137.50-187.50)	150.00 (112.50-150.00)	<0.001
Gn days (d)	11 (9-13)	10 (9-12)	0.004
Gn dose (U)	2100.00 (1537.50-2700.00)	1500.00 (1050.00-2175.00)	<0.001
Number of oocytes retrieved (n)	14 (8-20)	17 (11-25)	0.024
Fertilization methods, No. (%)			0.063
IVF	60.87% (56/92)	67.01% (65/97)	
ICSI	39.13% (36/92)	28.87% (28/97)	
IVF+ICSI	0.00% (0/92)	4.12% (4/97)	
Number of good-quality embryos (n)	5 (3-8)	7 (4-11)	0.014
Severe OHSS	1.09% (1/92)	4.12% (4/97)	0.194

BMI, body mass index; LH, luteinizing hormone; FSH, follicle stimulating hormone; T, testosterone; AMH, anti-Mullerian hormone; PCO, polycystic ovaries; GnRH, gonadotropin-releasing hormone; hCG, human chorionic gonadotropin; E_2_, estradiol; P, progesterone; Gn, gonadotropins; IVF, in vitro fertilization; ICSI, intracytoplasmic sperm injection; OHSS, ovarian hyperstimulation syndrome; SD, standard deviation; IQR, interquartile range.

Embryos were evaluated on the third day after fertilization. Good-quality embryos were all developed from 2 pronuclei zygotes and met the following criteria: (1) had more than 5 blastomeres; (2) size difference was less than 20%; (3) fragmentation was less than 50% (12).

The level of IL-6 was lower in the normal BMI group than in the high BMI group (*P* = 0.026). The levels of IL-1β, IL-2, IL-4, IL-9, lL-10, TNF-α and IFN γ were also lower in the normal BMI group than in the high BMI group, while IL-17, IL-12p70, G-CSF, GM-CSF and IFN-α were lower in the high BMI group, but the difference was not significant (**Table 2**).

**Table 2:** Levels of serum inflammatory factors in high BMI and normal BMI PCOS patients.

	High BMI (n=92)	Normal BMI (n=97)	*P*
	median (IQR)	median (IQR)	
IL-1β (pg/mL)	8.05 (1.52-14.94)	2.46 (0.01-12.27)	0.058
IL-2 (pg/mL)	3.23 (1.57-6.16)	3.07 (1.26-6.55)	0.706
IL-4 (pg/mL)	2.66 (1.73-7.08)	2.17 (0.98-5.10)	0.078
IL-6 (pg/mL)	12.16 (4.15-92.48)	7.08 (3.01-31.94)	0.026
IL-9 (pg/mL)	0.92 (0.64-1.21)	0.81 (0.58-1.09)	0.101
IL-10 (pg/mL)	2.85 (1.51-9.41)	2.78 (1.38-5.79)	0.438
IL-17 (pg/mL)	3.18 (2.25-10.31)	3.23 (2.05-6.01)	0.672
IL-12p70 (pg/mL)	2.32 (1.48-3.26)	2.60 (2.00-3.72)	0.059
TNF-α (pg/mL)	25.38 (5.41-167.18)	15.49 (2.26-161.59)	0.134
G-CSF (pg/mL)	2.38 (2.09-3.61)	2.46 (2.13-3.63)	0.744
GM-CSF (pg/mL)	1.61 (1.35-2.60)	1.74 (1.41-2.37)	0.921
IFN-α (pg/mL)	2.05 (1.37-5.02)	3.19 (1.57-4.68)	0.414
IFN γ (pg/mL)	9.36 (4.25-14.80)	7.93 (3.73-13.14)	0.353

BMI, body mass index; IL, interleukin; IFN, interferon; TNF, tumor necrosis factor; G-CSF, granulocyte colony stimulating factor; GM-CSF, granulocyte macrophage colony stimulating factor; IQR, interquartile range.

There were no significant differences between the high BMI and normal BMI groups in the pregnancy outcomes of the first transfer cycle (**Table 3**). However, the cumulative clinical pregnancy (55.67% *vs.* 36.96%, *P* = 0.010) and live birth rates (35.05% *vs.* 21.74%, *P* = 0.043) were higher in the normal BMI group.

**Table 3:** Pregnancy outcomes in PCOS patients.

	High BMI (n=92)	Normal BMI (n=97)	*P*
First transfer cycle clinical pregnancy (%)	31.52% (29/92)	45.36% (44/97)	0.051
First transfer cycle miscarriage (%)	44.83% (13/29)	36.36% (16/44)	0.470
First transfer cycle live birth (%)	17.39% (16/92)	28.87% (28/97)	0.062
Cumulative clinical pregnancy (%)	36.96% (34/92)	55.67% (54/97)	0.010
Cumulative miscarriage (%)	41.18% (14/34)	37.04% (20/54)	0.698
Cumulative live birth (%)	21.74% (20/92)	35.05% (34/97)	0.043

BMI, body mass index.

The first cycle transfer and cumulative pregnancy outcomes were shown in **Tables 4**, **5**. The levels of IL-2 and IL-10 were higher in the first cycle transfer live birth group (*P* = 0.033, *P* = 0.040). The level of GM-CSF was lower in the first cycle transfer and cumulative live birth group (*P* = 0.024, *P* = 0.028) and higher in the first cycle transfer and cumulative miscarriage group (*P* = 0.003, *P* < 0.001). Other serum inflammatory factors were not associated with the first cycle transfer and cumulative pregnancy outcomes.

**Table 4:** Expression of serum inflammatory factors and first cycle transfer pregnancy outcomes in PCOS patients.

	First cycle clinical pregnancy (n=73)	First cycle non-clinical pregnancy (n=116)	*P*	First cycle miscarriage (n=29)	First cycle non-miscarriage (n=160)	*P*	First cycle live birth (n=44)	First cycle non-live birth (n=145)	*P*
	median (IQR)	median (IQR)		median (IQR)	median (IQR)		median (IQR)	median (IQR)	
IL-1β (pg/mL)	8.28 (0.49-14.94)	4.57 (0.13-13.93)	0.485	2.70 (0.42-11.21)	10.51 (0.50-33.50)	0.276	10.51 (0.52-32.57)	4.14 (0.42-12.04)	0.161
IL-2 (pg/mL)	3.83 (1.60-6.92)	2.95 (1.40-5.72)	0.161	2.80 (1.32-5.82)	5.27 (1.92-7.40)	0.120	5.28 (1.99-7.34)	2.88 (1.40-5.72)	0.033
IL-6 (pg/mL)	7.45 (2.70-38.11)	9.39 (3.56-55.81)	0.196	6.25 (2.95-31.20)	8.34 (2.49-43.26)	0.572	8.34 (2.51-41.23)	9.37 (3.48-52.67)	0.569
IL-10 (pg/mL)	3.26 (1.59-9.05)	2.57 (1.42-4.96)	0.142	2.85 (1.29-6.09)	4.58 (2.00-10.00)	0.123	4.58 (2.04-9.97)	2.60 (1.42-5.14)	0.040
TNF-α (pg/mL)	12.02 (2.90-166.65)	19.63 (3.18-164.07)	0.628	8.70 (1.50-140.38)	20.43 (6.83-239.97)	0.097	20.43 (7.13-230.07)	18.64 (2.72-160.01)	0.377
G-CSF (pg/mL)	2.61 (2.16-3.96)	2.31 (2.08-3.33)	0.054	2.87 (2.23-4.00)	2.54 (2.05-4.01)	0.221	2.54 (2.05-3.92)	2.38 (2.11-3.63)	0.653
GM-CSF (pg/mL)	1.67 (1.35-2.40)	1.64 (1.40-2.60)	0.824	2.18 (1.58-2.92)	1.50 (1.31-2.05)	0.003	1.50 (1.31-2.05)	1.83 (1.41-2.70)	0.024

IL, interleukin; TNF, tumor necrosis factor; G-CSF, granulocyte colony stimulating factor; GM-CSF, granulocyte macrophage colony stimulating factor; IQR, interquartile range.

**Table 5:** Expression of serum inflammatory factors and cumulative pregnancy outcomes in PCOS patients.

	Cumulative clinical pregnancy (n=88)	Cumulative non-clinical pregnancy (n=101)	*P*	Cumulative miscarriage (n=34)	Cumulative non-miscarriage (n=155)	*P*	Cumulative live birth (n=54)	Cumulative non-live birth (n=135)	*P*
	median (IQR)	median (IQR)		median (IQR)	median (IQR)		median (IQR)	median (IQR)	
IL-1β (pg/mL)	7.44 (0.41-13.29)	5.00 (0.55-13.96)	0.957	2.70 (0.42-10.49)	8.24 (0.38-24.45)	0.390	8.24 (0.48-18.20)	4.57 (0.50-12.23)	0.435
IL-2 (pg/mL)	3.59 (1.60-6.82)	3.00 (1.40-5.56)	0.198	3.05 (1.36-6.24)	5.01 (1.77-6.95)	0.250	5.01 (1.85-6.92)	3.00 (1.40-5.66)	0.078
IL-6 (pg/mL)	7.12 (2.70-40.28)	10.66 (3.76-78.61)	0.062	6.70 (2.82-33.13)	7.28 (2.56-41.21)	0.706	7.280 (2.61-41.19)	9.88 (3.65-61.64)	0.295
IL-10 (pg/mL)	3.10 (1.39-8.90)	2.59 (1.50-4.94)	0.370	2.95 (1.37-7.23)	3.57 (1.41-9.30)	0.412	3.57 (1.48-9.29)	2.66 (1.46-5.29)	0.250
TNF-α (pg/mL)	12.02 (2.90-161.74)	25.22 (3.22-167.18)	0.346	10.09 (1.86-150.19)	14.00 (5.55-183.02)	0.259	14.01 (5.68-176.51)	18.88 (2.76-163.63)	0.836
G-CSF (pg/mL)	2.60 (2.13-4.13)	2.31 (2.10-3.10)	0.053	2.93 (2.27-4.51)	2.49 (2.03-3.85)	0.120	2.49 (2.04-3.77)	2.42 (2.12-3.63)	0.932
GM-CSF (pg/mL)	1.85 (1.36-2.64)	1.55 (1.39-2.40)	0.530	2.31 (1.82-3.17)	1.52 (1.30-2.10)	<0.001	1.52 (1.31-2.08)	1.83 (1.41-2.71)	0.028

IL, interleukin; TNF, tumor necrosis factor; G-CSF, granulocyte colony stimulating factor; GM-CSF, granulocyte macrophage colony stimulating factor; IQR, interquartile range.

After age, AMH and high BMI were adjusted, the number of oocytes retrieved and good-quality embryos were not correlated with high IL-6, high TNF-α, and high IL-1β (**Supplementary Table 1**).

Combined with the above statistical results and the clinical significance of serum cytokines, the impact of inflammatory factors on pregnancy outcomes was analyzed. With the factors of age, AMH and high BMI adjusted, high TNF-α was negatively correlated with the first cycle clinical pregnancy rate (Regression Coefficient, 0.201; 95%CI, 0.051-0.794; *P* = 0.022), and cumulative clinical pregnancy rate (Regression Coefficient, 0.130; 95%CI, 0.033-0.509; *P* = 0.003, **Table 6**). High IL-6 was negatively correlated with the cumulative live birth rate (Regression Coefficient, 0.185; 95%CI, 0.036-0.955; *P* = 0.044). However, high IL-1β was positively correlated with the live birth rate (Regression Coefficient, 7.785; 95%CI, 1.366-44.348; *P* = 0.021) and cumulative live birth rate (Regression Coefficient, 9.006; 95%CI, 1.579-51.350; *P* = 0.013).

**Table 6:** Logistic regression analysis of inflammatory factors associated with first cycle transfer and cumulative pregnancy outcomes with age and AMH adjusted.

	First cycle clinical pregnancy		First cycle live birth		Cumulative clinical pregnancy		Cumulative live birth	
	OR (95%CI)	*P*	OR (95%CI)	*P*	OR (95%CI)	*P*	OR (95%CI)	*P*
Age	1.080 (0.927-1.259)	0.323	1.233 (1.009-1.507)	0.041	1.001 (0.865-1.157)	0.992	1.113 (0.937-1.323)	0.223
AMH	0.944 (0.836-1.066)	0.351	0.925 (0.785-1.089)	0.348	0.920 (0.818-1.035)	0.165	0.890 (0.768-1.032)	0.124
High BMI	0.470 (0.168-1.317)	0.151	0.360 (0.092-1.411)	0.143	0.449 (0.165-1.219)	0.116	0.390 (0.119-1.285)	0.122
High TNF-α	0.201 (0.051-0.794)	0.022	0.456 (0.101-2.064)	0.308	0.130 (0.033-0.509)	0.003	0.287 (0.067-1.235)	0.094
High IL-6	1.298 (0.434-3.884)	0.641	0.364 (0.067-1.976)	0.242	0.823 (0.285-2.378)	0.719	0.185 (0.036-0.955)	0.044
High IL-1β	1.508 (0.443-5.133	0.511	7.785 (1.366-44.348)	0.021	1.558 (0.456-5.326	0.479	9.006 (1.579-51.350)	0.013

AMH, anti-Mullerian hormone; BMI, body mass index; TNF, tumor necrosis factor; IL, interleukin; High TNF-α: TNF-α >16.5pg/mL; High IL-6: IL-6 >5.4pg/mL; High IL-1β >12.4pg/mL.

## Discussion

Chronic inflammatory status in PCOS patients would affect normal ovarian function, including the synthesis and release of sexual hormones, follicle maturation, and ovulation. The mechanisms underlying the relationship between overweight and chronic inflammatory status were complicated and PCOS patients had a high degree of heterogeneity. The testing of inflammatory factors should be focused rather than generalizing, otherwise, it would waste medical resources and increase the cost of patient diagnosis and treatment. This was the first report analyzing the levels of inflammatory factors in PCOS patients with different BMI groups and effective predictors of IVF/ICSI pregnancy outcomes.

Patients with PCOS often had the characteristic of elevated AMH. In accordance with the study of Bansal P, et al. (13), which indicated AMH levels were significantly positively correlated with LH/FSH ratio, significantly higher LH/FSH and AMH levels were observed in normal BMI PCOS patients in our study. In a recent study (14), Pei Xu, et al. revealed that FSH receptor expression was decreased in parallel with BMI, whereas the E_2_ level on the hCG trigger day was significantly lower, and this was caused by a dysfunctional insulin pathway. Our study confirmed that the hCG day E_2_ was lower while Gn days and dose were longer and higher in the high BMI group. In addition, the high BMI group had lower levels of AMH and fewer oocytes retrieved. In line with Wendy Vitek, et al.’s study, women with obesity had lower AMH levels, and fewer oocytes retrieved than women with normal BMI (15).

Obesity was a metabolic disease characterized by a chronic inflammatory state, with patients having high levels of proinflammatory cytokines, chemokines, and markers of oxidative stress (16). In the screening of numerous inflammatory factors, the project should be focused rather than blind, especially IL-1β, IL-6, TNF-α and GM-CSF. The level of inflammatory factors like IL-1β correlated with obesity of PCOS patients, and PCOS patients who carried T allele of *IL-1β* gene promoter region (-511) were at high risk of obesity (17). This was in line with our study, which showed that the level of IL-6 and IL-1β were significantly elevated in high BMI PCOS patients. There were already studies that represented an important link between the metabolic performance of PCOS and chronic inflammation. TNF-α, IL-1β, and IL-6 released by adipose tissue resident macrophages could lead to insulin resistance (18). Insulin resistance, hyperandrogenism, chronic low-grade inflammation, adipose tissue hypertrophy, and dysfunction might interact in a vicious circle in the pathophysiology of PCOS (19).

As we already known, AMH was regarded as the remark of ovarian reserve and influences pregnancy outcomes. The higher baseline AMH level in PCOS women resulted in a lower live birth rate, clinical pregnancy rate, and fertilization rate but did not influence the cumulative live birth rate (20). After age and AMH were adjusted, our study showed high TNF-α was negatively correlated with pregnancy outcomes. A recent study illustrated that, especially in obese PCOS patients, the level of reactive oxygen species was high (21), which triggered an inflammatory chain reaction that activates TNF-α and NF-κB, and increased the risk of oocyte aneuploidy and damage oocytes. Lan Y, et al. showed significantly higher TNF-α were observed in the fetal arrest group than in the live birth group (22). On the contrary, Khalid M Salama, et al. detected the concentrations of IL-1β and TNF-α in endometrial secretion at the time of oocyte retrieval were higher in the pregnant group than in the non-pregnant group significantly (23). In their study, the embryo transfer was all fresh cycle, and our study paid more attention to the first transfer cycle and cumulative pregnancy outcomes among PCOS patients, which was different. In addition, considering the factor of high BMI, our study showed high levels of inflammatory factors still had the influence on pregnancy outcomes. Thus, the great impact of inflammatory factors on pregnancy outcomes should also be noticed in normal BMI PCOS patients.

Compared to healthy controls, PCOS patients presented with a systemic chronic low degree of inflammation during pregnancy, a finding significantly associated with the risk of adverse obstetric outcomes (24). Currently, clinical studies explored the potential role of IL-6 in human oocyte maturation and subsequent embryonic development, but reliable conclusions could not be drawn based on these results (25). In our study, high IL-6 was negatively correlated with the cumulative live birth rate of PCOS patients. Other studies showed that high levels of IL-6 in the follicular fluid were beneficial for oocyte maturation and were associated with increased clinical pregnancy and embryo implantation rates (26). Further studies were needed to illustrate the influence of IL-6 on pregnancy outcomes. Our study showed high IL-1β was positively correlated with the first cycle transfer and cumulative live birth rate of PCOS patients. This might be explained by IL-1β playing dual roles in implantation and embryogenesis. It inhibited blastocyst attachment and stimulates placental growth, but might be toxic to embryos at very high concentrations (27).

A progressive and statistically significant decreasd in the live birth rate and an increased in the miscarriage rate were observed in obese patients with PCOS (28). In addition, some studies reported that obesity was an important risk factor for adverse pregnancy outcomes in PCOS patients (29). The high inflammatory state of the uterus in PCOS could be the cause of severe pregnancy complications, ranging from miscarriage to placental dysfunction (30). TNF-α and G-CSF were the key cytokines involved in the mechanism of decreased embryo development potential in PCOS metabolic syndrome patients (31). Elaimi et al. found that the proportion of chromosomal abnormalities in mouse blastocysts increased in culture media containing GM-CSF (32). GM-CSF had not been found to have a positive effect on improving miscarriage caused by aneuploidy in elderly women (33). However, some trials with human embryos showed that supplementing GM-CSF could improve the pregnancy rate of patients with multiple IVF failures and previous miscarriages (34). Cochrane Gynaecology and Fertility Group reported that whether the administration of G‐CSF and GM-CSF improved ongoing pregnancy or overall clinical pregnancy rates or reduces miscarriage rate compared to no treatment or placebo were uncertain (35, 36). The level of GM-CSF was significantly higher in both first cycle transfer and cumulative miscarriage groups in our study. However, G‐CSF and GM-CSF did not influence pregnancy outcomes of PCOS patients, and combining clinical experience, these factors were not included in the final logistic regression. Further studies of GM-CSF influencing PCOS patients’ miscarriage rates were needed to clarify the mechanisms.

There were some limitations in our study. First, since obese (BMI > 28 kg/m^2^) women only occupied a small amount of PCOS patients in our study, we chose BMI 24 kg/m^2^ as the criteria to separate PCOS patients into different groups in our study. Whether there was a greater impact of chronic inflammatory status on pregnancy outcomes in obese PCOS patients required further analysis. Second, our study could not completely rule out the bias in retrospective data collection. Lastly, our study did not separately investigate the influence of inflammatory factors on fresh and frozen cycle pregnancy outcomes, because a number of PCOS patients could not achieve fresh cycle transfer due to the risk of ovarian hyperstimulation syndrome. More research data could be included and find the relationship between serum cytokines and other factors which influence miscarriage rates. However, chronic inflammatory status in patients with PCOS had a long-term impact on the body, making it more meaningful to analyze the first transfer cycle, which patients cared about most, and cumulative pregnancy outcomes, more data could be added to make the research more complete.

## Conclusion

In summary, the study revealed that high BMI had a negative effect on pregnancy outcomes among PCOS patients. For PCOS patients, in addition to BMI, attention should also be paid to inflammatory indicators. The testing of inflammatory factors should be focused rather than generalizing. High TNF-α and IL-6 were negatively correlated with the pregnancy outcomes, but high IL-1β was positively correlated with the live birth rates. PCOS patients with high GM-CSF need to be vigilant about miscarriage.

## Data availability statement

The raw data supporting the conclusions of this article will be made available by the authors, without undue reservation.

## Ethics statement

The study was approved by the ethics committee of the reproductive center of the Third Hospital of Peking University, No. 2019SZ-041.

## Author contributions

YH, SY and RL developed the conception of the study and all authors contributed to the research discussion. YH, JY, YW and LC took part in patients follow-up and contributed to the data analysis. YH wrote the initial draft of the paper and all authors contributed to manuscript revision. All authors contributed to the article and approved the submitted version.
