# Supplementary material for: Influencing of serum inflammatory factors on IVF/ICSI outcomes among PCOS patients with different BMI
Source: Front Endocrinol (Lausanne). 2023 Aug 24;14:1204623. doi: 10.3389/fendo.2023.1204623 (PMC10484569; doi:10.3389/fendo.2023.1204623)
Supplement: Supplementary file 1 [file Table_1.docx]

Supplementary Table 1 Linear regression analysis of the number of oocytes retrieved and good-quality embryos in PCOS patients.

|  | Number of oocytes retrieved | | Number of good-quality embryos | |
| --- | --- | --- | --- | --- |
|  | Regression Coefficient (95%CI) | *P* | Regression Coefficient (95%CI) | *P* |
| Age | 0.330 (-0.289-0.948) | 0.292 | 0.344 (-0.014-0.703) | 0.059 |
| AMH | 0.653 (0.163-1.143) | 0.010 | -0.039 (-0.323-0.245) | 0.784 |
| High BMI | -2.714 (-6.888-1.460) | 0.200 | -2.339 (-4.758-0.080) | 0.058 |
| High IL-6 | -2.436 (-7.064-2.191) | 0.298 | -1.343 (-4.025-1.338) | 0.322 |
| High TNF-α | -2.791 (-7.269-1.688) | 0.219 | -1.470 (-4.066-1.125) | 0.263 |
| High IL-1β | 1.628 (-3.632-6.888) | 0.540 | 0.353 (-2.695-3.401) | 0.818 |

AMH, anti-Mullerian hormone; BMI, body mass index; IL, interleukin; TNF, tumor necrosis factor; High IL-6: IL-6 >5.4pg/mL; High TNF-α: TNF-α >16.5pg/mL; High IL-1β >12.4pg/mL
